# Guidelines and Diagnostic Assays for *Mycoplasma genitalium*

**DOI:** 10.1093/ofid/ofag003

**Published:** 2026-03-30

**Authors:** Caleb M Ardizzone, Stephen J Jordan

**Affiliations:** Department of Medicine, Division of Infectious Diseases, Indiana University School of Medicine, Indianapolis, Indiana, USA; Department of Microbiology and Immunology, Indiana University School of Medicine, Indianapolis, Indiana, USA; Department of Medicine, Division of Infectious Diseases, Indiana University School of Medicine, Indianapolis, Indiana, USA; Department of Microbiology and Immunology, Indiana University School of Medicine, Indianapolis, Indiana, USA

Perhaps no sexually transmitted infection (STI) causes more confusion for clinicians when considering whether testing is clinically indicated than *Mycoplasma genitalium*. This is, in part, because outside of sexual health clinics, *M. genitalium* is still relatively unknown to many clinicians and because management of *M. genitalium* infections is more complex than other STIs. National and international guidelines differ in their recommendations for *M. genitalium* testing in specific syndromes and symptoms (eg, some guidelines recommend testing in acute syndromes, others only in persistent or recurrent syndromes). These differences result from multiple contributing factors. First, different regions have varied access to *M. genitalium* testing. Compared to *Neisseria gonorrhoeae* and *Chlamydia trachomatis*, *M. genitalium* nucleic acid amplification tests (NAATs) were only very recently available (in the United States, the first *M. genitalium* NAAT received FDA clearance in 2019), and access to *M. genitalium* testing continues to vary widely by region; in 2021, <15% of US public health labs offered *M. genitalium* testing [1]. Outside the United States and Canada, commercially available *M. genitalium* NAATs, including tests for macrolide resistance, are generally more available in some regions, and healthcare plans are more likely to cover *M. genitalium* testing. As a result, the degree of access to *M. genitalium* testing (including resistance testing) is an important factor that impacts guideline recommendations (ie, testing is more broadly recommended in regions with readily available and inexpensive *M. genitalium* testing). Second, compared to *N. gonorrhoeae* and *C. trachomatis*, much less is known about the association of *M. genitalium* with anogenital syndromes, especially reproductive tract morbidity, and data confirming that *M. genitalium* treatment reduces risk for these sequelae are less robust, especially in asymptomatic individuals. These associations are further complicated by the fact that co-infection of *M. genitalium* with other STIs, especially *N. gonorrhoeae* and *C. trachomatis*, is common in certain populations [2, 3]. As a result, the contribution of *M. genitalium* infection to acute anogenital syndromes and sequelae in mixed infections is unclear. Third, unlike *N. gonorrhoeae* and *C. trachomatis*, the decision to treat *M. genitalium* infection may carry increased cost and risk for adverse effects at the individual level and selection of antibiotic-resistance at the community level [4]. *M. genitalium* is intrinsically resistant to beta-lactam antibiotics, and available treatment options are limited. Optimal antibiotic stewardship practices that slow the development of drug-resistant infections are paramount. Given *M. genitalium*'s small genome, single nucleotide polymorphisms (SNPs) readily confer high-level drug resistance [5]. Indeed, SNPs at position 2058 or 2059 in the single rRNA operon confer high-level macrolide resistance and increasing resistance to fluoroquinolones is a growing concern [6]. Lastly, guidelines have different update cadences (ie, the US CDC STI guidelines historically have been updated every 5–7 years). As a result, as additional evidence emerges about the public health impact of *M. genitalium* infections and *M. genitalium* NAATs become more accessible, updated recommendations may converge with those from more recently updated versions. In summary, guidelines consider these factors and interpret these limited data through the lens of their unique public health resources, perceived benefit from testing, and risk for antimicrobial resistance. Clinicians should test for *M. genitalium* as directed by their regional guidelines and after considering the downstream effects that treating a positive result may have on the patient and community (reviewed by Manhart et al [7]).

Here, we will briefly discuss the available *M. genitalium* assays and then review the 2021 US Centers for Disease Control and Prevention (CDC) STI treatment guideline recommendations for *M. genitalium* testing [8]. Given the international readership of *Open Forum Infectious Diseases*, we will also mention assays available outside the United States and compare recommendations for *M. genitalium* testing in guidelines from Canada [9], United Kingdom (BASHH) [10], Europe [11], and Australia [12].

## ASSAYS TO DETECT *M. GENITALIUM* INFECTION

### Culture and Serological Assays

Historically considered the gold standard assay to confirm an active STI, in vitro cultivation of STIs has been replaced by highly sensitive and specific NAATs. Although in vitro culture is still used in specific circumstances (eg, antimicrobial resistance testing of *N. gonorrhoeae* and *T. vaginalis*), in vitro cultivation of *M. genitalium* is not recommended as it is technically difficult and in vitro expansion takes weeks to months due to the organism's extremely slow growth rate. Outside of research laboratories, in vitro *M. genitalium* culture has no clinical utility. Similarly, serological assays against *M. genitalium* are not recommended as they have lower sensitivity and specificity than NAATs and frequently cross-react with other *Mycoplasma* species, especially *M. pneumoniae* [13].

### Non-rapid Nucleic Acid Amplification Tests

Laboratory-based, high-throughput NAATs remain the primary diagnostic platforms for detecting *M. genitalium* in urogenital specimens (Table 1); no extra-genital NAAT has Food and Drug Administration (FDA) clearance for *M. genitalium*. Nucleic acid amplification tests can be separated into non-rapid NAATs (result in 2–5 days) and rapid NAATs (result in <90 min, often available within the clinical encounter). Rapid NAATs for *M. genitalium* have only recently become available. Advantages of the non-rapid NAATs are very high sensitivity and specificity and the wide number of specimen types that can be used for testing. A key limitation is that the delay in time to result prevents same-encounter, pathogen-specific treatment. A key advantage of rapid NAATs is that the result is available within the clinical encounter to guide the need for *M. genitalium* treatment. Disadvantages are potentially lower sensitivity and cost.

**Table 1.** US FDA-Cleared Molecular *M. genitalium* Assays

Manufacturer	Assay	Platform	Regulatory Status	Pathogen(s) Detected	*M. genitalium* Target(s)	Detection Method(s)	Recommended Specimen(s)	Other Validated Specimen(s)	FDA Decision Number
Hologic, Inc. (Marlborough, MA)	Aptima^®^ *Mycoplasma genitalium*	Panther^®^ System	FDA-cleared, De Novo	*M. genitalium*	rRNA	TC, TMA, HPA	Male: urine	Male: urethral swab, penile meatal swab	DEN180047
							Female: vaginal swab	Female: urine, endocervical swab	
Roche Molecular Systems, Inc. (Pleasanton, CA)	cobas^®^ TV/MG	cobas^®^ 6800/8800 System	FDA-cleared, 510(k)	*M. genitalium*, *T. vaginalis*	DNA	PCR	Male: urine	Male: penile meatal swab	K190433
							Female: vaginal swab	Female: urine, endocervical swab	
Abbott Molecular, Inc. (Des Plaines, IL)	Alinity™ m STI	Alinity™ m System	FDA-cleared, 510(k)	*M. genitalium*, *T. vaginalis*, *N. gonorrhoeae*, *C. trachomatis*	rRNA	PCR	Male: urine	Male: none	K202977
							Female: vaginal swab	Female: endocervical swab	
Roche Molecular Systems, Inc. (Pleasanton, CA)	cobas^®^ liat CT/NG/MG	cobas^®^ liat System	FDA-cleared, 510(k), CLIA-waived	*M. genitalium*, *N. gonorrhoeae*, *C. trachomatis*	DNA, rRNA	PCR	Male: urine	Male: none	K240197
							Female: vaginal swab	Female: none	

Partially adapted from Waites et al [22].

Abbreviations: FDA, Food and Drug Administration; PCR, polymerase chain reaction; rRNA, ribosomal ribonucleic acid; DNA, deoxyribonucleic acid; TC, target capture; TMA, transcription-mediated amplification; HPA, hybridization protection assay; MG, *Mycoplasma genitalium*; TV, *Trichomonas vaginalis*; NG, *Neisseria gonorrhoeae*; CT, *Chlamydia trachomatis*.

In the United States, the first non-rapid *M. genitalium* NAAT to obtain FDA authorization was the Aptima® *Mycoplasma genitalium* test (Hologic, Marlborough, MA) and was granted De Novo classification in 2019 (DEN180047) [14]. This established the regulatory pathway for subsequent assays cleared through the 510(k) process. This assay uses transcription-mediated amplification of *M. genitalium* rRNA on the fully automated Panther® platform. The assay is validated for clinician- or self-collected vaginal swabs, endocervical swabs, male and female urine, clinician-collected male urethral swabs, and self-collected penile meatal swabs. The FDA labeling designates vaginal swabs as the preferred specimen for women and urine for men and recommends retesting with a vaginal swab if suspicion remains after a negative result from a non-preferred specimen. Analytical validation demonstrated limits of detection (LOD) approximately 10 genome equivalents per reaction, high reproducibility across runs and instruments, and no cross-reactivity with other urogenital mycoplasmas [15]. Clinical performance was evaluated in the multicenter AMES trial, which reported 90.9%–98.9% sensitivity and 98%–99.4% specificity for recommended specimen types and 77.8%–98.2% sensitivity and 97.8%–99.6% specificity for non-preferred specimens [16].

Later in 2019, the cobas® TV/MG NAAT (Roche Molecular Systems, Pleasanton, CA) received FDA clearance (K190433) for dual detection of both *T. vaginalis* and *M. genitalium* [17]. The assay detects DNA from both pathogens simultaneously using real-time PCR on the cobas® 6800/8800 systems. The assay is validated for clinician- or self-collected vaginal swabs, endocervical swabs, male and female urine, and clinician- or self-collected penile meatal swabs for *M. genitalium*. The FDA labeling designates vaginal swabs as the preferred specimen for women and urine for men and recommends retesting with a preferred specimen type if *M. genitalium* is strongly suspected after a negative test result using a non-preferred specimen. The LOD ranges from 0.5–4 genome copies/ml for *M. genitalium* depending on specimen type and test strain. A multicenter study demonstrated comparable diagnostic performance to the Aptima® assay, reporting 96.6%–100% sensitivity and 97.0%–97.6% specificity for preferred specimen types and 83.1%–86.4% sensitivity and 97.0%–98.4% specificity for non-preferred sample types [18].

In 2022, the Alinity™ m STI multiplex assay (Abbott Molecular, Des Plaines, IL) received FDA clearance (K202977), expanding multiplex *M. genitalium* NAATs on a single specimen to include *C. trachomatis*, *N. gonorrhoeae*, and *T. vaginalis* [19]. For *M. genitalium*, the assay is validated for clinician- or self-collected vaginal swabs, endocervical swabs, and male urine. The FDA labeling designates vaginal swabs as the preferred specimen for women and urine for men. The assay claims a LOD of 165 genome equivalents/mL for *M. genitalium*, although one study reported a higher LOD [20]. A small clinical evaluation showed the assay to be in 86.4% agreement with the cobas® TV/MG [21].

Outside the United States, numerous molecular assays for *M. genitalium* detection are available under the CE-IVD marking (reviewed by Waites et al [22]), although none are currently cleared by the US FDA. Only the assays discussed above hold both CE-IVD and FDA clearance; all other CE-marked tests remain restricted to non-US markets.

### Rapid Nucleic Acid Amplification Tests

In 2025, the cobas® liat CT/NG/MG (Roche Molecular Systems, Pleasanton, CA) received FDA clearance and a CLIA waiver (K240197), becoming the first point-of-care (POC) *M. genitalium* test for use by non-laboratory personnel in the United States. The assay detects *C. trachomatis*, *N. gonorrhoeae*, and *M. genitalium* via dual targets (*mgpC* DNA and 23S rRNA) within approximately 20 min [23]. The assay is validated for clinician- or self-collected vaginal swabs and male urine. A recently published multicenter study provided comprehensive clinical performance data for the assay, reporting sensitivities of 95.2% in clinician- or self-collected vaginal swabs and 97.1% in male urine and specificities of 97.8%–99.2% across validated specimen types [24]. Performance did not differ between symptomatic and asymptomatic individuals or between trained laboratory staff and POC operators [24]. As a CLIA-waived assay, it can be used for testing in non-laboratory settings, such as urgent care clinics and sexual health centers, facilitating same-visit diagnosis and treatment, since results can be delivered at the POC.

### Antibiotic Resistance Tests

Macrolide and fluoroquinolone resistance in *M. genitalium* remains a major clinical concern, with macrolide resistance rates exceeding 40%–50% in some US cohorts and quinolone resistance emerging [6, 25]. In the United States, no commercially available macrolide-resistance assay yet exists, though multiple assays are in development. Instead, resistance testing is limited to laboratory-developed or research-use-only assays performed in reference laboratories [26]. These assays typically use targeted PCR and sequencing of the 23S rRNA gene to detect mutations associated with macrolide resistance (A2058T/G/G, A2059G) and of *parC* and *gyrA* genes for fluoroquinolone resistance. Outside the United States, assays like the ResistancePlus MG (SpeeDx, Australia), Allplex MG & AziR (Seegene, South Korea), and VIASURE macrolide resistance-associated mutations (23S rRNA) kit (Certest Biotec, Spain) enable simultaneous detection of *M. genitalium* and resistance-associated 23S mutations (A2058T/G/G, A2059G), supporting resistance-guided sequential therapy [27]. The lack of FDA-cleared resistance assays remains a key limitation that must be addressed.

## GUIDELINE RECOMMENDATIONS FOR *M. GENITALIUM* TESTING

### *M. genitalium* Testing in Asymptomatic Individuals

With the exception of *M. genitalium* contacts or in limited circumstances, all guidelines recommend against *M. genitalium* testing in asymptomatic individuals (Table 2). Instead, testing should be restricted to symptomatic individuals presenting with either acute, persistent, or recurrent syndromes in which *M. genitalium* is strongly suspected (ie, persistent symptoms despite treatment for *N. gonorrhoeae* and *C. trachomatis*). There is a lack of evidence that asymptomatic *M. genitalium* infections poses a risk for reproductive tract sequelae (ie, pelvic inflammatory disease [PID], infertility, maternal-fetal complications, etc.) in women, and data in men is even more sparse [7]. *M. genitalium* has not been associated with infertility [28]. Also, although few studies exist on the natural history of *M. genitalium*, the majority of asymptomatic infections appear to spontaneously resolve (ie, organism clearance without antibiotics) within a year [29].

**Table 2.** National Guideline Recommendations for *M. genitalium* Testing

	US CDC	BASHH (UK)	European	Australian	PHAC (Canada)
Release date	2021	2025	2021	2024	2022
Asymptomatic individuals	No	No	No	No	No
Urogenital syndromes					
Acute NGU	No	Yes	Yes, includes all urethritis	Yes	No
Acute mucopurulent cervicitis	No	No	Yes	Yes	No
Persistent or recurrent NGU	Yes	Yes	Yes	Yes	Yes, if already treated for NG/CT and tested negative for NG/CT
Persistent or recurrent cervicitis	Yes	Yes, especially if post-coital bleeding	Yes	Yes	Yes, if post-treatment for NG/CT and NG/CT NAAT negative
Epididymo-orchitis	No	Yes, if symptoms persist, other STIs ruled out, and suspicion is high	Yes, if age < 50 y	No	No
Pelvic inflammatory disease	Consider	Yes	Yes	Yes	Yes, if symptomatic post-treatment for NG/CT and NG/CT NAAT negative
Other symptoms	No recommendations	Not recommended for nonspecific symptoms, unless persistent and MG suspected	Test for acute pelvic pain, intermenstrual/post-coital bleeding, pathogen-negative dysuria	Test for post-coital bleeding	No recommendations
Extragenital syndromes					
Proctitis	Consider if persistent symptoms after treatment	Yes, if symptoms persist, other STIs ruled out, and suspicion is high	Yes, once NG and CT excluded	Consider if MG contact or other STIs ruled out	No
Oropharyngeal	No	No	No	No	No
Prostatitis	No	No	No	No	No
Other considerations					
Macrolide resistance	Yes, if available	Yes	Yes	Yes	Testing possible through National Microbiology Lab
Quinolone resistance	No commercially available tests	Consider for treatment failures	Consider for treatment failures	Consider for treatment failures, but has variable positive predictive value	Not routinely, but possible through National Microbiology Lab
Test-of-cure (TOC)	TOC not recommended if asymptomatic, unless azithromycin was used and resistance testing not performed; retest ≥3 wks after completing treatment	TOC recommended if (1) symptom present or (2) persistent infection suspected	Consider TOC in all patients; retest ≥3 wks after completing treatment	If (1) symptoms present or (2) risk of reinfection/sequelae, consider TOC 2–3 wks after completing treatment	TOC recommended if (1) symptoms present or (2) lives in region with high resistance; retest ≥3 wks after completing treatment
Sex partners	Consider testing. If MG NAAT+, consider treating to prevent index case reinfection	Test only ongoing sex partners	Test only ongoing sex partners	Test only ongoing sex partners	Partner testing not routinely recommended. Consider treating partner, regardless of symptoms
Pregnancy	Same as nonpregnant women	Same as nonpregnant women	Same as nonpregnant women. Consider testing before terminating pregnancy	Same as nonpregnant women	no recommendation (insufficient data)
Persons living with HIV	Same as persons not living with HIV	Same as persons not living with HIV	Same as persons not living with HIV	Same as persons not living with HIV	Same as persons not living with HIV

Abbreviations: BASHH, British Association for Sexual Health and HIV; PHAC, Public Health Agency of Canada; NGU, nongonococcal urethritis; TOC, test-of-cure; NAAT, nucleic acid amplification test; MSM, men who have sex with men; MG, *M. genitalium*; NG, *Neisseria gonorrhoeae*; CT, *Chlamydia trachomatis*; HIV, human immunodeficiency virus; STI, sexually transmitted infection.

### Lower Genital Tract Syndromes (Cervicitis and Urethritis)

In acute nongonococcal urethritis (NGU) and mucopurulent cervicitis, US and Canadian guidelines recommend against routine testing for *M. genitalium* infection. Instead, *M. genitalium* testing should be restricted to persistent or recurrent urethritis and cervicitis, when *M. genitalium* infection is suspected (in Canada, after *C. trachomatis* and *N. gonorrhoeae* have been excluded). This approach reflects antimicrobial-resistance and antibiotic-stewardship concerns and limited access to *M. genitalium* testing—particularly resistance testing in the United States—which could otherwise increase fluoroquinolone use. In contrast, European and Australian guidelines recommend testing for *M. genitalium* more broadly in symptomatic syndromes, including symptom-based indications (eg, post-coital bleeding). BASHH guidelines recommend *M. genitalium* testing in NGU and only in persistent cervicitis if other infections have been ruled out and suspicion remains high (not at initial acute presentation).

### Upper Genital Tract Syndromes (PID, Epididymitis, and Prostatitis)

Data are conflicted on the association of *M. genitalium* with acute PID [30, 31]. In US guidelines, testing for *M. genitalium* may be considered in acute PID. If testing is performed, resistance testing should also be included, if available. Other guidelines recommend *M. genitalium* testing in all individuals with PID symptoms/syndrome in combination with macrolide-resistance testing, if available. Canadian guidelines recommend testing only persistently symptomatic individuals who test negative for *N. gonorrhoeae* and *C. trachomatis*. Due to a lack of evidence, testing for *M. genitalium* in prostatitis is not recommended and testing in acute epididymo-orchitis is not recommended in the US, Canada, and Australia [32, 33]. BASHH and European guidelines recommend testing in persistent epididymo-orchitis despite treatment and in men with acute epididymo-orchitis if < 50 years old, respectively.

### Extragenital Infections

Extragenital testing for *M. genitalium* infection is generally not recommended, except in persistent proctitis or when other STIs have been excluded. Oropharyngeal infection with *M. genitalium* is uncommon and considered unlikely to transmit *M. genitalium* to partners [34]. Therefore, guidelines recommend against testing for *M. genitalium* infection as part of routine screening or in symptomatic individuals. Asymptomatic rectal *M. genitalium* infections are common, especially in men who have sex with men, and the clinical significance of these infections remains unclear [35]. Therefore, guidelines recommend against routine testing for rectal *M. genitalium* infections. In acute proctitis, the association with *M. genitalium* is unclear, and *M. genitalium* testing is either not recommended (United States, Canada) or only recommended when other causes have been excluded (BASHH, Europe, Australia); US guidelines consider testing for persistent proctitis. In sexually acquired reactive arthritis, case reports have identified *M. genitalium* as a possible cause, but there are no data that supports *M. genitalium* testing [36].

### Recommended Specimen Types for *M. genitalium* Testing

Given their superior sensitivity and specificity, guidelines recommend NAATs to detect *M. genitalium* infection. *M. genitalium* NAATs have been US FDA-cleared for use on urine, urethral, penile meatal, endocervical, and vaginal swabs; no NAAT is FDA-cleared for oral or rectal testing. US CDC and Canadian guidelines do not list a preferred specimen for genital testing. The BASHH, European, and Australian guidelines recommend urine as the preferred specimen in men and either a self-collected or clinician-collected vaginal swab in women. For individuals who have undergone vaginoplasty or phalloplasty as part of gender-affirming care, the optimal specimen type remains unknown.

### Specific Populations

#### Pregnancy

Guidelines recommend against routine screening for *M. genitalium* in pregnancy as no clear association has been found between *M. genitalium* infection and adverse outcomes in pregnancy [37]. Further, treating *M. genitalium* infection in pregnancy introduces additional complexity as only azithromycin is considered safe throughout pregnancy [38 ]. As rates of macrolide-resistant *M. genitalium* may be >50% in some settings, it is possible that no safe treatment option exists. Due to the lack of safety data in humans and animal studies showing fetal harm, most guidelines list moxifloxacin as contraindicated in pregnancy. However, in cases of persistent cervicitis or PID during pregnancy when *M. genitalium* infection is suspected, testing for *M. genitalium* may be considered after considering the risk in consultation with an infectious diseases specialist. In cases of macrolide-resistant *M. genitalium* infection during pregnancy, the optimal approach is unknown. Given the lack of strong evidence of maternal-fetal complications from *M. genitalium* and possible risk from moxifloxacin, delaying treatment in asymptomatic individuals until after delivery is often recommended.

#### Sex Partners and Other Populations

Testing sex partners of confirmed individuals with *M. genitalium* infection should be considered with the goal of preventing reinfection of the index patient. US guidelines state that partner testing can be considered; Canadian guidelines do not recommend testing but instead consider treating the partner. BASHH, European, and Australian guidelines specify that only ongoing sex partners need testing. Unlike *N. gonorrhoeae* and *C. trachomatis* in which empiric partner treatment is indicated, partner treatment for *M. genitalium* should be restricted to only partners who test positive for *M. genitalium* or in partners that cannot be tested. Testing recommendations are not different in men who have sex with men or persons living with HIV.

### Resistance Testing and Cure Confirmation Testing

Given the significant variation in access to antibiotic resistance testing, guidelines differ on the recommendation for macrolide and quinolone resistance testing. When available, testing for macrolide resistance is recommended as the result informs antibiotic choice as part of sequential therapy (ie, doxycycline followed by either high-dose azithromycin when a macrolide-susceptible infection is confirmed or moxifloxacin in macrolide-resistant infections or when resistance testing is unavailable). Quinolone testing is not commonly performed in the United States but could be considered outside the United States in cases of treatment failure, especially to moxifloxacin. US guidelines recommend a *M. genitalium* test-of-cure (TOC) only when azithromycin has been used and the status of macrolide susceptibility is unknown. Most other guidelines recommend a TOC when treatment failure or reinfection is considered possible; European guidelines recommend a universal TOC. To prevent false positives due to detecting residual nucleic acids from non-viable organisms, most guidelines recommend delaying a TOC until at least 3 weeks after completing antibiotics.

## CONCLUSION

Indications for *M. genitalium* testing in specific syndromes vary across guidelines, but all guidelines recommend that testing be restricted to symptomatic individuals or contacts (except Canadian guidelines). In the United States, *M. genitalium* testing should only be performed on urogenital specimens, as routine oropharyngeal and rectal testing are not currently recommended. Also, testing should be restricted to persistent or recurrent urethritis and cervicitis and may be considered in cases of PID. Other international guidelines extend testing to include acute syndromes and specific symptoms. All guidelines recommend that NAATs be used to detect *M. genitalium* infection. Aligning with best antibiotic stewardship practices, sexual partners of individuals with *M. genitalium* infection should be tested first and treated only if positive (Canadian guidelines are an exception) or if testing is unavailable. In summary, determining when to test for *M. genitalium* can be challenging for clinicians, but clinicians should be familiar with their governing guideline recommendations. Following these recommendations helps ensure that patients are only tested for *M. genitalium* when clinically indicated and that both the patient and community derive the maximal benefit from appropriate antibiotic stewardship practices.
